# Effect of dioxins on regulation of tyrosine hydroxylase gene expression by aryl hydrocarbon receptor: a neurotoxicology study

**DOI:** 10.1186/1476-069X-8-24

**Published:** 2009-06-06

**Authors:** Eiichi Akahoshi, Seiko Yoshimura, Saeko Uruno, Mitsuko Ishihara-Sugano

**Affiliations:** 1 Functional Material Laboratory, Corporate Research & Development Center, Toshiba Corporation, 1 Komukai-Toshiba cho, Saiwai-ku, Kawasaki 212-8582, Japan

## Abstract

**Background:**

Dioxins and related compounds are suspected of causing neurological disruption. Epidemiological studies indicated that exposure to these compounds caused neurodevelopmental disturbances such as learning disability and attention deficit hyperactivity disorder, which are thought to be closely related to dopaminergic dysfunction. Although the molecular mechanism of their actions has not been fully investigated, a major participant in the process is aryl hydrocarbon receptor (AhR). This study focused on the effect of 2, 3, 7, 8-tetrachlorodibenzo-p-dioxin (TCDD) exposure on the regulation of TH, a rate-limiting enzyme of dopamine synthesis, gene expression by AhR.

**Methods:**

N2a-Rβ cells were established by transfecting murine neuroblastoma Neuro2a with the rat AhR cDNA. TH expression induced by TCDD was assessed by RT-PCR and Western blotting. Participation of AhR in TCDD-induced TH gene expression was confirmed by suppressing AhR expression using the siRNA method. Catecholamines including dopamine were measured by high-performance liquid chromatography. A reporter gene assay was used to identify regulatory motifs in the promoter region of TH gene. Binding of AhR with the regulatory motif was confirmed by an electrophoretic mobility shift assay (EMSA).

**Results:**

Induction of TH by TCDD through AhR activation was detected at mRNA and protein levels. Induced TH protein was functional and its expression increased dopamine synthesis. The reporter gene assay and EMSA indicated that AhR directly regulated TH gene expression. Regulatory sequence called aryl hydrocarbon receptor responsive element III (AHRE-III) was identified upstream of the TH gene from -285 bp to -167 bp. Under TCDD exposure, an AhR complex was bound to AHRE-III as well as the xenobiotic response element (XRE), though AHRE-III was not identical to XRE, the conventional AhR-binding motif.

**Conclusion:**

Our results suggest TCDD directly regulate the dopamine system by TH gene transactivation via an AhR-AHRE-III-mediated pathway. The AhR- mediated pathway could have a particular AhR-mediated genomic control pathway transmitting the effects of TCDD action to target cells in the development of dopaminergic disabilities.

## Background

Halogenated aromatic hydrocarbons (HAHs) such as polychlorinated biphenyls (PCBs) and poly-chlorodibenzo-*p*-dioxins (PCDDs) affect human health when they are absorbed by the body. Their effects are predominantly negative, such as oncogenesis, reproductive toxicity, immunosuppression, and neurological dysfunction [1-4]. One of the dioxins, 2, 3, 7, 8-tetrachlorodibenzo-p-dioxin (TCDD), leads to neurobehavioral abnormalities associated with both cognitive and locomotor systems [5,6]. While the precise anatomical regions and cell types targeted by TCDD are largely unidentified, the functional abnormalities may be consistent with findings of improper brain maturation in epidemiological and experimental animal studies. Experimental animal studies have indicated that perinatal exposure to TCDD has a marked effect on learning ability in rats and monkeys [7-9]. Epidemiological studies have suggested that children accidentally exposed to PCB and dioxins exhibit delayed motor development and show a tendency to hyperactivity [10]. Another report has indicated an association of serum concentrations of dioxins with the prevalence of learning disability and attention deficit hyperactivity disorder (ADHD) [11]. However, the precise mechanisms of TCDD action in the brain have not been fully elucidated.

A major participant in the process of dioxin toxicity is the aryl hydrocarbon receptor (AhR) [12,13]. AhR is a ligand-activated transcription factor belonging to the basic helix-loop-helix/Per-Arnt-Sim (bHLH-PAS) transcription factor superfamily [14,15]. Dioxins and PCBs, which are the major ligands of AhR, bind and activate it. The ligand-activated AhR translocates into the nucleus with the aryl hydrocarbon receptor nuclear translocator (ARNT), and binds to the xenobiotic response element (XRE) on the target gene enhancer, thereby activating gene expression [16,17]. The genes of many xenobiotic-metabolizing enzymes possess XRE and are activated by the AhR-ARNT complex. Phase I enzymes including cytochrome P_450 _CYP1A1 or CYP1B1 and phase II enzymes such as glutathione S-transferase (GST), metabolize xenobiotics in the process of detoxification [18-21].

Whereas our understanding of the molecular mechanisms by which TCDD modulates gene regulation is progressing, results reported on the neurotoxicity of AhR are not fully consistent with the current understanding. TCDD has been recently shown to induce CYP1A1 mRNA and protein via the AhR-ARNT complex in granule cells of the rat cerebellum [22]. Although AhR is expressed in various regions of the brain, CYP1A1 expression in response to TCDD has been observed in a few brain cells, and the expression of phase II enzymes such as GST in response to TCDD has not been observed in any region [23,24]. It is difficult to explain AhR-mediated neurotoxicity in response to TCDD or related HAHs based only on the previously reported molecular mechanisms. There may also be mechanisms that are independent of xenobiotic metabolism and detoxification. Molecular mechanisms connecting AhR-mediated gene expression in neuronal cells and TCDD-induced neurotoxic effects, including behavioral abnormalities, should therefore be explored.

We have attempted to identify a target gene of AhR-mediated neurotoxicity in neurons. To analyze the relationship between AhR activation and neurotoxicity in a previous study, we stably transfected cells of the murine neuroblastoma cell line, Neuro2a, with AhR cDNA to produce N2a-R cells. These cells exhibited overexpression and functional activation of AhR. Using N2a-R cells, we demonstrated that tyrosine hydroxylase (TH) mRNA level increased and that this increase correlated to AhR activation [25].

TH is the rate-limiting enzyme of dopamine synthesis and a selective marker of catecholaminergic neurons [26]. Because abnormal expression of TH is related to dopaminergic dysfunction such as in Parkinson's disease [27], TH may be a key molecule in dopaminergic function. According to epidemiological and experimental animal studies, dioxins and related HAHs are potentially able to induce hyperactivity and learning disability [9,10]. Behavioral abnormalities including ADHD are thought to be closely related to dopaminergic neural function. Evidence supporting dopaminergic dysfunction in ADHD derives from three research areas: the neuropharmacology of stimulant medication [28], the behavior and biochemistry of animal models [29], and neuroimaging studies in adults with ADHD [30,31]. Many molecular genetic studies have focused on genes participating in the dopamine system. TH is hyperexpressed in the Naples high-excitability (NHE) rat, which shows behavioral traits resembling the main aspects of ADHD in humans [32,33]. If behavioral abnormalities induced by TCDD are attributable to dopaminergic dysfunction, TH is an important candidate for a key molecule of TCDD-induced neurotoxic effects. In fact, another study has shown that the level of dopamine was associated with subchronic exposure of TCDD in rats [34]. Although overproduction of dopamine in the hippocampus and brainstem was observed in rats exposed to TCDD, there is no direct evidence indicating that the activation of AhR by TCDD causes abnormal activation of dopamine synthesis.

In the present study, we analyzed the regulation of the TH gene by TCDD-activated AhR. It will help in the development of treatment to prevent behavioral abnormality caused by exogenous chemicals. We describe the transcriptional activation of the TH gene depending on the AhR differing from the generally recognized XRE-mediated pathway.

## Methods

### Dioxin treatment

TCDD was purchased from Kanto Chemical (Tokyo, Japan) and was maintained as a stock solution (50 μg/ml) in dimethyl sulfoxide (DMSO). Cells were treated by adding TCDD to the culture medium.

### Cell cultures

Neuro2a, a murine neuroblastoma cell line, was purchased from the American Type Culture Collection. Several N2a-R cell lines were established by transfecting Neuro2a cells with the rat AhR cDNA, as previously reported [25]. N2a-Rα and N2a-Rβ are clones obtained during the establishment of Neuro2a cell lines and are maintained as stable transfectants. The N2a-Vc cell line was also established by transfecting Neuro2a cells with an intact vector [25]. These cells were routinely grown in Dulbecco's modified Eagle's medium and medium F12 in a 1:1 ratio, supplemented with 10% fetal calf serum. Cells were grown at 37°C in a humidified atmosphere of 5% CO_2_/95% air.

### Western blot analysis

Cells were collected and homogenized in phosphate-buffered saline. Total protein concentrations were determined using the Bio-Rad protein assay (Bio-Rad Laboratories Inc, California, USA). Proteins (30 μg/lane) were separated on 10% SDS-PAGE and transferred to PVDF membranes (Millipore, Massachusetts, USA). Membranes were blocked with Block Ace (Dainippon Seiyaku, Osaka, Japan) and incubated with primary antibody. AhR and TH were detected using an anti-AhR mouse monoclonal antibody (1:500; Affinity BioReagents, Colorado, USA) and an anti-TH mouse monoclonal antibody (1:500; Affinity BioReagents), respectively. After incubation with the primary antibody, the membranes were washed three times with Tris-buffered saline (TBS) and incubated with a rabbit alkaline phosphatase-conjugated anti-mouse IgG (1:1000; Bio-Rad Laboratories Inc.). The blots were visualized with BCIP-NBT substrate solution (Kirkegaard & Perry Laboratories, Inc., Maryland, USA).

### Reverse transcription: RT-PCR

Total RNA was isolated from the cells using RNeasy (QIAGEN, Hilden, Germany) and reverse transcribed with Superscript II reverse transcriptase (Invitrogen, California USA). PCR was performed with Ex Taq polymerase (Takara Bio Inc., Shiga, Japan) using the following primers: AhR, 5'-CCGTCCATCCTGGAAATTCGAACC-3' and 5'-CCTTCTTCATCCGTTAGCGGTCTC-3'; TH, 5'-CCACGGTGTACTGGTTCACT-3' and 5'-GGCATAGTTCCTGAGCTTGT-3'; CYP1A1, 5'-TTGCCCTTCATTGGTCACAT-3' and 5'-GAGCAGCTCTTGGTCATCAT-3'; and cyclophilin, 5'-GTCTCCTTCGAGCTGTTTGCAG-3' and 5'-CCATCCAGCCATTCAGTCTTGG-3'. PCR products were electrophoresed on 3% Nusieve 3:1 agarose gel (Takara Bio Inc.) and stained with ethidium bromide.

### RNA interference

siRNAs targeting the mouse AhR gene (si-AhR) and a scrambled siRNA (scramble) were synthesized (QIAGEN).These siRNAs were transiently transfected into the cells using RNAiFect (QIAGEN) according to the manufacturer's instructions. Sequences of the siRNAs were as follows: si-AhR, 5'-UCCCACAUCCGCAUGAUUATT-3' and 5'-TTAGGGUGUAGGCGUACUAAU-3'; scramble, 5'-UACCCAUCAUGACCCUGAUTT-3' and 5'-TTAUGGGUAGUACUGGGACUA-3'. After transfection, the cells were cultured in growth medium overnight, exposed to 10 nM TCDD for 24 h, and then collected. Total RNAs were extracted from the cells and reverse-transcribed as described for reverse transcription (RT)-PCR protocol. The resulting cDNAs were used in real-time PCR as templates.

### Real-time PCR

Real-time PCR was performed with DNA Engine (Bio-Rad Laboratories Inc.) using the DyNamo SYBR Green qPCR Kit (Finnzymes, Oy, Finland). The primers were the same as those described for RT-PCR. The PCR conditions were as follows: 95°C for 5 min, 40 cycles of 95°C for 10 s, 58°C for 10 s, and 72°C for 20 s, with fluorescence measurements read during each cycle. The expression levels of AhR and TH mRNAs were normalized to those of cyclophilin mRNA.

### High-performance liquid chromatography (HPLC)

The amounts of L-dopa and catecholamines (dopamine, noradrenaline, and adrenaline) were determined using a fluorescent HPLC method by SRL (Tokyo, Japan). Briefly, the cells were collected and homogenized in 60% HClO_4_(2.0 × 10^7 ^cells/ml). After centrifugating the homogenates, the supernatants were collected, derivatized with ethylenediamine to detect L-dopa or with diphenylethylenediamine to detect catecholamines, and then analyzed by HPLC using a fluorescent detector.

### Immunohistochemistry

Formalin fixed paraffin embedded sections (6 μm thick) of rat brain were obtained from Genostaff (Tokyo, Japan). The sections were dewaxed and pretreated by microwaving for 10 min in citrate buffer, pH 6.0. To detect AhR protein, the sections were treated with 3% hydrogen peroxide in methanol for 15 min and 10% goat serum for 30 min, and then incubated with anti-AhR mouse monoclonal antibody (1:2500; Affinity BioReagents) overnight. After washing with TBS, the sections were incubated with Histofine Simple Stain MAX PO (M) (Nichirei, Tokyo, Japan), washed with TBS, and then visualized by DAB/H_2_O_2_. To detect TH protein, the sections were treated with 10% goat serum for 30 min and incubated with anti-TH rabbit polyclonal antibody (1:400; Millipore) overnight. After washing with TBS, the sections were treated with Biotin blocking system (Dako, Glostrup, Denmark), incubated with a biotin-conjugated goat anti-rabbit IgG (1:400; Dako), and then washed with TBS. Then, the sections were treated with AP-conjugated streptavidin, washed with TBS, and then visualized using ALP Red (Diagnostic Biosystems, CA, USA). For double staining, the sections were first stained with anti-AhR antibody followed by staining with anti-TH antibody. The sections were counterstained with Mayer's hematoxylin and mounted with Malinol (Mutoh Chemical Co., Tokyo, Japan).

### Construction of plasmids

Five successive fragments of the mouse TH gene 5'-flanking region (-488 to +30 bp) were generated by PCR using mouse genomic DNA as a template (Promega, WI, USA). PCRs were performed with five distinct forward primers and a common reverse primer, resulting in the generation of five successive deletion constructs (-488 to +30, -375 to +30, -285 to +30, -167 to +30, and -65 to +30 bp). Each primer was designed to contain an additional restriction site (*Kpn*I or *Nhe*I) to facilitate directional cloning. The primer sequences were as follows: forward 1 (-488 to -463 bp), 5'-cgtggtaccACATACACTGGGGCAGTGAGTAGAT-3'; forward 2 (-375 to -350 bp), 5 '-cggggtaccAGATTTATTTGTCTCCAAGGGCTAT-3'; forward 3 (-285 to -260 bp), 5'-cggggtaccATTAGAGAGCTCTAGATGTCTCCTG-3'; forward 4 (-167 to -142 bp), 5'-cccggtaccCTAATGGGACGGAGGCCTCTCTCGT-3'; forward 5 (-65 to -40 bp), 5'-cggggtaccGTGGGGGACCCAGAGGGGCTTTGAC-3'; and reverse (+5 to +30 bp), 5'-gcagctagcAAGCTGGTGGTCCCGAGTTCTGTCT-3'. Lower case letters in the sequence indicate the restriction sites added at the 5' end of each primer. The deletion fragments were cloned into the *Kpn*I/*Nhe*I site in a luciferase reporter vector PGV-B2 (Toyo-B-Net Co. Ltd., Tokyo, Japan).

### Luciferase reporter gene assay

Neuro2a cells (0.8 × 10^5^) were cultured overnight in 24-well plates. The next day, the cells were transiently cotransfected with each reporter plasmid containing the five distinct deletion fragments, pcDNA-rAhR, and pcDNA4/V5-His/lacZ at a ratio of 2:1:1 using Lipofectamine 2000(Invitrogen) according to the manufacturer's instructions. The plasmid pcDNA4-rAhr expressing rat AhR was constructed previously [25]. The plasmid pcDNA/V5-His/LacZ (Invitrogen) expressing β-galactosidase was used as a transfection efficiency control. Twenty-four hours after transfection, the cells were exposed to DMSO or 10 nM TCDD for 24 h and then collected. Luciferase assays were performed using the PicaGene LT2.0 kit (Toyo-B-Net Co. Ltd.) according to the manufacturer's instructions. The luciferase activities were measured using a Mithras LB940 luminometer (Berthold Technologies, Bad Wildbad, Germany). Luciferase activity was normalized with β-galactosidase activity, based on the results of control experiments by using of β-galactosidase and Renilla Luc. (data not shown).

### Electrophoretic mobility shift assay (EMSA)

AhR and ARNT were generated by *in vitro *coupled transcription and translation using the *TNT*-coupled rabbit reticulocyte lysate system according to the manufacturer's instructions (Promega). Double-stranded oligonucleotide probes were synthesized and labeled with Alexa Fluor 532 (Invitrogen). EMSA was performed using Gel Shift Kit, second generation (Roche Diagnostics K.K, Tokyo, Japan) according to the manufacturer's instructions. Equal volumes of AhR and ARNT were premixed in the presence of DMSO or 1 μM TCDD for 2 h at 37°C. The premixes were incubated with labeled probes at final concentrations of 1.2 fmol/μl or 4.5 fmol/μl in the binding buffer containing poly (dI-dC) at the final concentration of 8 ng/μl for 15 min at 15°C. A competition assay was performed by adding 200-fold molar excess of unlabeled oligonucleotide probes to the reaction. The DNA-protein complexes were resolved on 6% denaturing polyacrylamide gel in 1× Tris-borate/EDTA buffer. Subsequently, the gels were scanned using a fluorescent scanner (Typhoon 8600; GE Healthcare Bio-Sciences Corp., New Jersey, USA). The sequences of the oligonucleotide probes were as follows: C (-238 to -213 bp region of the murine TH gene), 5'-TGTCTTCATGTCGTGTCTAGGGCGG-3'; XRE (-1029 to -1004 bp of the murine CYP1A1 gene), 5'-CCTCCAGGCTCTTCT**CACGC**AACTCC-3'. The consensus sequence of XRE is in bold.

### Statistics

All values are presented as mean ± standard deviation (SD). All experiments were performed at least three times. Data were analyzed by Student's *t*-test using the Microsoft Excel software package.

## Results

### TCDD induced TH gene expression based on AhR activation

**Figure 1:**
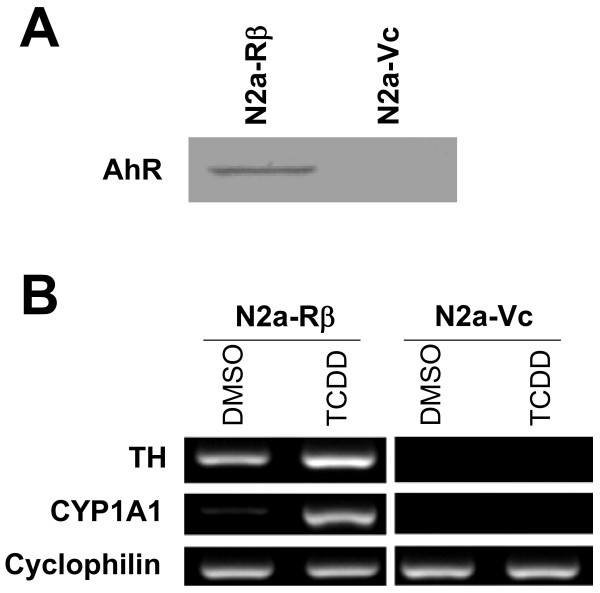
**Induction of TH gene by TCDD exposure inN2a-Rβ cells**. (A) AhR protein (96 kDa) in N2a-Rβ cells was detected by Western blotting using an anti-AhR antibody. No signal was detected in N2a-Vc cells. (B) The expression of TH and CYP1A1 mRNAs in N2a-Rβ cells was detected by RT-PCR. TH mRNA expression was induced in addition to CYP1A1 mRNA in TCDD-exposed N2a-Rβ cells. CYP1A1, a typical XRE-mediated gene, was used as a marker of AhR activation. TH and CYP1A1 mRNAs were not detected in N2a-Vc cells. Data represent triplicate experiments.

In order to develop an experimental model and analyze the relationship between AhR activation and neurotoxicity, we constructed the N2a-R cell line, which is an AhR-overexpressing cell line constructed by transfection of Neuro2a cells with the rat AhR cDNA [25]. From the N2a-R cell line, we obtained several clones containing N2a-Rα and N2a-Rβ cells. In a previous report, we demonstrated that N2a-Rα cells show hyperexpression of the TH gene caused by AhR activation in a ligand-independent manner. For further investigation of neurotoxic phenotype including TH gene expression by ligands, we used another clone regarded as N2a-Rβ cells. N2a-Rβ cells express a large amount of AhR, which acts in a ligand-dependent manner [25]. Figure 1A shows AhR protein expression in N2a-Rβ cells. AhR was clearly detected in N2a-Rβ cells by Western blot analysis, but was not detected in the control cells (N2a-Vc), which were transfected with the intact vector.

We performed RT-PCR analysis with the total RNA from TCDD-treated and untreated N2a-Rβ cells in order to analyze the ligand dependency of exogenous AhR in N2a-Rβ cells. We chose mRNA for CYP1A1, a typical AhR-mediated gene, as a functional marker of AhR activation. CYP1A1 mRNA was detected with specific primers in the total RNA prepared from the TCDD treated-N2a-Rβ cells, and was weakly detected in TCDD-untreated (DMSO) N2a-Rβ cells (Fig. 1B). CYP1A1 mRNA was not detected in the control cells (N2a-Vc). These results suggest that the highly expressed exogenous AhR protein of N2a-Rβ cells was functional and ligand-activated.

RT-PCR analysis was also performed with specific primers for TH mRNA with the total RNA prepared from TCDD-treated and -untreated N2a-Rβ cells. Fig. 1B shows the expression of TH mRNA in N2a-Rβ and N2a-Vc cells. Expression of the TH gene was weak in TCDD-untreated (DMSO) N2a-Rβ cells. It was elevated when the N2a-Rβ cells were treated with TCDD, as compared to the TCDD-untreated N2a-Rβ cells. TH gene expression was detected to a very negligible extent in N2a-Vc cells. These results support the acceleration of TH gene expression by AhR in a ligand-dependent manner.

**Figure 2:**
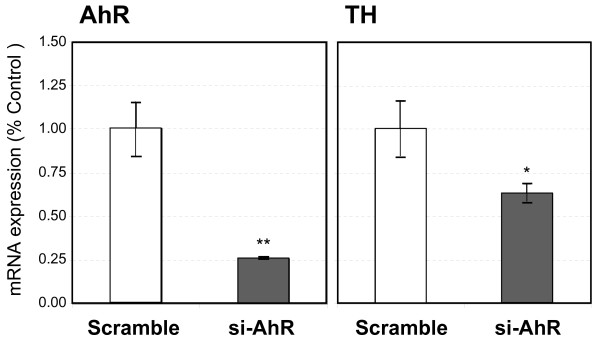
**Suppression of TH mRNA expression by si-AhR (siRNA against AhR)**. N2a-Rβ cells were transiently transfected with si-AhR or scrambled siRNA (Scramble) and then exposed to 10 nM TCDD. Twenty-four hours after TCDD exposure, total RNA extracted from transfected cells was subjected to real-time quantitative PCR. Expression levels of AhR mRNA (left panel) and TH mRNA (right panel) were significantly suppressed by si-AhR. Results are normalized to the expression level of cyclophilin mRNA and are expressed as the ratio of si-AhR to scramble. Data are presented as mean ± SD of four replicate determinations.**P < 0.01 and *P < 0.05.

To confirm TH gene induction by the AhR-mediated pathway in the presence of TCDD, we attempted to suppress transcription of the AhR gene in N2a-Rβ cells using RNA interference. The siRNAs, si-AhR and scramble, were separately transfected into N2a-Rβ cells. Two days after transfection, the expression of AhR and TH mRNA was measured by real-time quantitative PCR. AhR mRNA was decreased by about 25% in N2a-Rβ cells transfected with si-AhR, and the TH mRNA level was decreased by about 55%. The expression levels of the AhR and TH genes were not affected by scramble siRNA (Fig. 2). These results clearly indicate that the increase in TH gene expression was induced through the AhR-mediated pathway in N2a-Rβ cells.

### Effect of TCDD on dopamine synthesis

**Figure 3:**
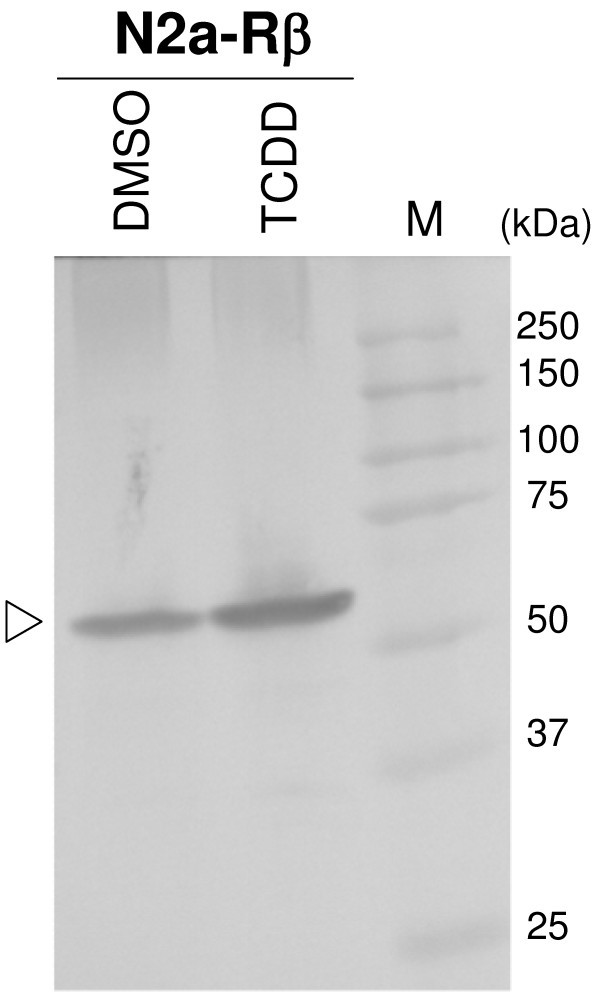
**Induction of TH protein by TCDD exposure inN2a-Rβ cells**. TH protein (56 kDa) in N2a-Rβ cells was detected by Western blotting using an anti-TH antibody (open triangle). The expression of TH protein was increased in TCDD-exposed N2a-Rβ cells as compared to DMSO. Lane M shows molecular weight markers (kDa). Data represent triplicate experiments.

TH protein is expressed and functional even if abnormal TH gene expression induced by TCDD is involved in neurotoxicity, such as abnormality of the dopamine system. We performed Western blot analysis with a specific antibody recognizing TH protein to evaluate the level of expression of TH protein in TCDD-treated N2a-Rβ cells. As shown in Fig. 3, the expression of TH protein was clearly elevated in the presence of TCDD. This result is consistent with the TH mRNA data in Fig. 1B.

The functional activity of TH was analyzed by measuring intermediary products in catecholamine biosynthesis. We measured the amounts of L-dopa, dopamine, noradrenaline, and adrenaline in TCDD-treated N2a-Rβ cells by high performance liquid chromatography. TH is the rate-limiting enzyme of dopamine synthesis, catalyzing the production of L-dopa from tyrosine. If the expression of TH is increased, the amount of L-dopa should increase proportionally. The amounts of L-dopa and dopamine were increased 1.75- and 1.59-fold, respectively, by TCDD exposure, whereas the amount of noradrenaline was not substantially changed. Adrenaline was not detected either in the presence or the absence of TCDD (Table 1).

**Table 1:** Increase in dopamine level by TCDD exposure to N2a-Rβ cells

L-dopa and catecholamines	Amount		Fold increase
		
	DMSO	TCDD	
L-dopa (ng/2 × 10^7^cells)	118.8 ± 18.9	208.8 ± 18.0	1.75**
Dopamine (ng/2 × 10^7^cells)	17.7 ± 2.7	28.1 ± 2.9	1.59**
Noradrenaline (pg/2 × 10^7^cells)	66.8 ± 11.1	71.5 ± 8.6	1.07
Adrenaline (pg/2 × 10^7^cells)	N.D.	N.D.	N.D.

L-dopa and catecholamines in N2a-Rβ cells were measured by a fluorescent HPLC method. Amounts of L-dopa and dopamine were significantly higher in TCDD-exposed N2a-Rβ cells as compared to unexposed N2a-Rβ cells. Fold increase represents the ratios of the amounts in TCDD-exposed cells to those in unexposed cells. No change was observed in the amount of noradrenaline with or without TCDD exposure to N2a-Rβ cells.

Values are mean ± SD of four replicate determinations. ND, not detected. **P < 0.01.

### Colocalization of AhR with TH in midbrain

**Figure 4:**
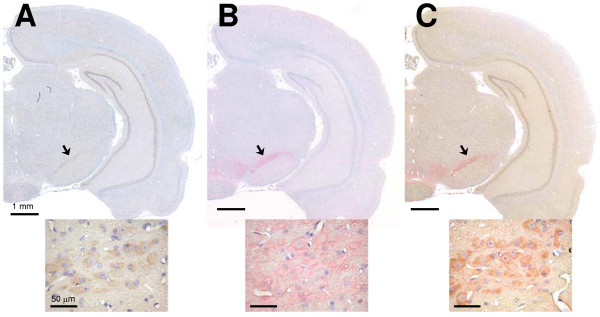
**Localization of AhR and TH proteins in the rat brain**. Immunohistochemical staining with anti-AhR and/or TH antibodies was performed on formalin fixed paraffin embedded coronal brain sections prepared from 10-week-old rats. Signals of AhR and TH proteins were developed using DAB/H2O2 (light brown) and ALP-RED (light red), respectively. Sections were counterstained with hematoxylin. (A) AhR protein was detected in the substantia nigra pars compacta (SNc) in the midbrain (black arrow), hippocampus, and dentate gyrus. Bar = 1 mm. (B) TH protein was detected in the SNc (black arrow) and hypothalamic area. Bar = 1 mm. (C) Double staining revealed colocalization of AhR and TH proteins in SNc (black arrow). Bar = 1 mm. Magnified (× 400) views of the SNc region were inset in each panel. Bar = 50 μm.

In our *in vitro *experimental model, treatment of N2a-Rβ cells with TCDD induced functional TH protein expression and elevated dopamine synthesis in a ligand-dependent manner. If these responses play an important role in the neurotoxicity of TCDD, AhR and TH protein should be expressed concomitantly in dopaminergic neurons in the brain. Therefore, we studied the colocalization of AhR and TH proteins in adult rat brain, *in vivo*, by immunohistochemical staining of the formalin fixed paraffin embedded coronal brain sections prepared from 10-week-old rats. AhR protein was detected in the substantia nigra pars compacta (SNc) of the midbrain, CA1–CA3 region of the hippocampus, and the dentate gyrus (Fig. 4A). SNc contains a major population of dopaminergic neurons, and TH protein, which is a marker of dopaminergic neurons, was mainly detected in SNc. In addition, antibody against TH protein stained the periaqueductal gray matter and supramammillary nucleus (Fig. 4B). These regions were reported previously as containing dopaminergic neurons [35,36]. To confirm the expression of AhR in the dopaminergic neurons, double staining with anti-TH antibody (red) and anti-AhR antibody (brown) was performed on the same tissue section (Fig. 4C). The high-magnification (× 400) image in Fig. 4C shows that AhR and TH proteins were colocalized in the SNc. AhR protein was expressed concomitantly with TH protein in a specific region of the brain that included the SNc. These results suggest that TCDD could influence the elevation of dopamine level through an action on AhR in the regions of the midbrain where AhR and TH proteins were detected.

### AhR-mediated transcriptional regulation of the TH gene

The finding that TCDD increased TH gene expression in N2a-Rβ cells makes it likely that AhR mediates this process. AhR is involved in xenobiotic metabolism and detoxification of exogenous chemicals such as dioxins, including TCDD, and related chemicals, as a ligand-activated transcriptional factor. In previous studies, the ligand-activated AhR bound the XRE motif on the enhancer of the target gene and induced the expression of XRE-mediated xenobiotic enzyme genes, including cytochrome P_450 _CYP1A1, CYP1B1, and GST [18-21]. The typical XRE motifs were not reported, however, in the identified transcriptional regulatory region of the TH gene.

**Figure 5:**
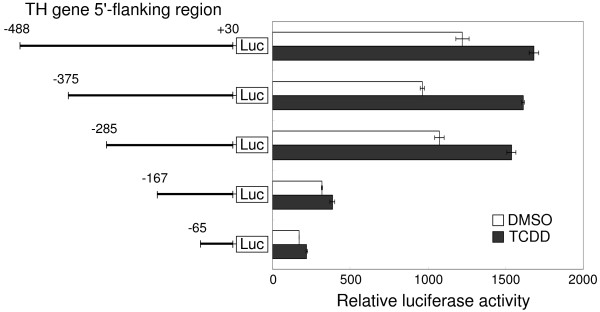
**TCDD-responsive region within the 5'-flanking region of the TH gene**. A deletion construct containing the 5'-flanking region of the murine TH gene fused to luciferase gene was generated. On the left is a schematic representation of the deletion constructs. Numbers on each construct indicate distance in bp from a putative transcription start site of the TH gene (+1). These constructs were transiently cotransfected into Neuro2a cells with an AhR expression vector (pcDNA4-rAhR) and a β-galactosidase expression vector (pcDNA4/V5-His/LacZ). The luciferase assay was performed 24 h after exposure to 10 nM TCDD. Relative luciferase activities were normalized with β-galactosidase activities. The 5'-flanking regions of the TH gene shorter than -167 bp did not increase luciferase activity in TCDD-exposed Neuro2a cells. Data are presented as mean ± SD of three replicate determinations.

To identify the regulatory region of AhR in the promoter region of the TH gene, we attempted to construct a reporter gene containing the upstream region of the TH gene. To search for a TCDD-responsive element in the TH promoter, 488 bp of the 5'-flanking sequence of the mouse TH gene was cloned. A series of plasmids were constructed containing different lengths of TH upstream sequences fused to the luciferase gene as a reporter gene (Fig. 5). These plasmids were transiently co-transfected with rat AhR-expressing plasmids and β-galactosidase-expressing plasmids as a transfection efficiency control into intact Neuro2a cells. To investigate the responses of TCDD corresponding to each region, we measured the luciferase activity of cells transfected with plasmids containing different lengths of TH upstream sequences. Transfectants with three plasmids containing 5'-flanking sequences longer than 285 bp showed significant luciferase activity in response to TCDD (Fig. 5). In contrast, transfectants with two plasmids containing less than 167 bp of the 5'-flanking sequence did not induce transcription of the luciferase gene in response to TCDD. From these results, the TCDD-responsive element appeared be localized in a sequence between -285 bp to -167 bp. The sequence contains several putative and confirmed *cis*-acting regulatory elements such as hypoxia-inducible factor-1, AP-1, CRE, E-box, and the partial dyad element [37,38]. However, the sequence does not contain XRE. If TCDD-activated AhR binds the sequence and affects the rate of transcription, AhR should regulate the transcription of the TH gene in an XRE-independent manner.

**Figure 6:**
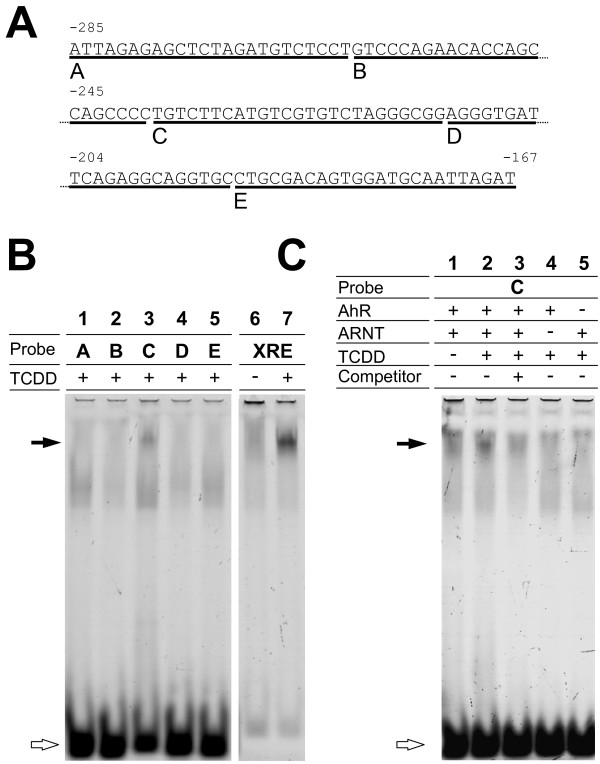
**Binding of the AhR-ARNT complex to the TCDD-responsive sequence in the TH enhancer**. The sequence is obtained from the murine TH gene 5'-flanking region (-285 to -167 bp). Each of the five underlined sequences (sequences A-E) was used as a TH 5'-flanking oligonucleotide probe for EMSA. (B) Alexa Fluor 532-labeled probes (sequences A-E) were incubated with the components indicated above each lane. EMSA signals were detected using a Typhoon 8600 fluorescent scanner. Black arrow indicates specific signal from the DNA-protein complex. In the presence of TCDD, the AhR-ARNT complex bound to sequence C (lane 3). (C) Using Alexa Fluor 532-labeled sequence C, EMSA was performed with and without unlabeled XRE probe as a competitor. In the presence of ARNT, the AhR-ARNT complex bound to sequence C (lane 1). This signal increased on exposure to 1 μM TCDD (lane 2) and decreased in the presence of unlabeled XRE as a competitor (lane 3). Data represent triplicate experiments.

To determine whether the sequence from -285 bp to -167 bp could induce the TH gene on exposure to TCDD, EMSA experiments were performed against the candidate sequence. Because TCDD-activated AhR translocates into the nucleus with ARNT and binds to DNA, EMSA was performed in the presence of AhR and ARNT generated *in vitro *(see Methods section). The candidate sequence, 5' flanking sequence of the TH gene (-285 to -167 bp), was divided into five fragments namely A, B, C, D, and E. We determined the sequences, which bound the AhR-ARNT complex on exposure to TCDD. Fig. 6B shows that the AhR-ARNT complex bound to sequence C (lane 3). To confirm whether the binding of the AhR-ARNT complex to sequence C is unique, competitive EMSA was performed using unlabeled XRE, a typical AhR-ARNT binding element, as a competitor. As shown in Fig. 6C, formation of the DNA-protein complex was inhibited by the XRE competitor (lane 3). Because formation of the complex was not observed in the absence of AhR and/or ARNT (lanes 4 and 5), the binding of TCDD-activated AhR to sequence C required ARNT, whereas sequence C did not contain the consensus XRE sequence (GCGTG). Therefore, it is likely that the elevation of TH gene expression induced by TCDD in neural cells is regulated by a pathway other than the AhR-XRE-mediated pathway that is used in the xenobiotic response.

## Discussion

Dioxins and related compounds cause diverse toxic responses, including hepatocellular damage, immune system disorders, reproductive system disorders, teratogenesis, carcinogenesis, and nervous system disorders [1-4]. The mechanism of actions of dioxins and related compounds involves genomic control to a large extent such as transcriptional activation. Abnormal expression of many TCDD-responsive genes is thought to be involved in the toxic effects of TCDD [16,17].

In a previous study, we reported that the TH gene is a TCDD-responsive gene in neurons [25]. In this study, we showed that TCDD not only increases TH gene expression but also the amounts of dopamine and L-dopa in TCDD-exposed murine neuroblastoma cells (Neuro2a) (Table 1). The result of these *in vitro *experiments indicates that TCDD can disturb the equilibrium of the dopamine system through abnormal dopamine synthesis resulting from abnormal TH gene expression.

Experimental animal studies and epidemiological studies indicated that TCDD and related compounds potentially cause neurodevelopmental disabilities such as learning disabilities and ADHD [10,11]. Clinical studies indicated that neurodevelopmental disabilities such as ADHD are closely related to the dopamine system of the midbrain. A PET study indicated that accumulation of [^18^F] dopa in the right midbrain was 48% greater in 10 children with ADHD as compared to 10 normal children [39]. Another PET study indicated that the [^11^C] dopa influx rate in the midbrain was 17.2% lower in 8 male adolescents with ADHD as compared to 8 normal boys [40]. A disorder of the dopamine system of midbrain is believed to be involved in ADHD.

The SNc, a midbrain nucleus, has abundant dopaminergic neurons, mainly A9 neurons [35,41]. As shown in Fig. 4, AhR, a dioxin receptor, was expressed in TH-positive neurons in the SNc, as determined by immunohistochemistry. This indicates that dopaminergic neurons in the SNc can be directly affected by TCDD. Briefly, it is likely that TCDD could interfere with the adjustment of dopamine concentration following the transactivation of the TH gene, and consequently disturb dopamine metabolism and/or dopaminergic neurotransmission of SNc. Therefore, we concluded that transactivation of the TH gene is one of the first molecular events of neurodevelopmental disabilities after exposure to TCDD.

At the molecular level, dioxins, including TCDD, affect gene transcription through AhR binding and successive AhR activation. AhR is a nuclear receptor that binds to dioxins and is a ligand-dependent transcription factor belonging to the bHLH-PAS family [14,15]. The mechanism of gene expression regulated by dioxins is not fully understood but is usually assumed to be attributable to the AhR-XRE-mediated reaction [16,17]. Many TCDD-responsive genes, including those encoding phase I and II enzymes, are upregulated through XRE in the promoter region, to which TCDD-activated AhR binds together with ARNT. However, we did not detect definite participation of XRE-driven transactivation in the effect of TCDD on TH gene expression.

Transcriptional regulation of the rat TH gene has been well studied. Basal transcription depends on transcription factor activity at the partial dyad element (-17 bp), CRE (-45 bp), AP-1(-205 bp), and the dyad symmetry element (-201 bp) [37,38]. Transcription is regulated by activity at CRE, AP-1, and hypoxia response element 1 (HRE-1, -225 bp). We analyzed transcriptional activity induced by TCDD in the TH promoter by deletion analysis and demonstrated a region at -285 bp to -167 bp in the 5'-flanking sequence of the TH gene that had transcriptional activity stimulated by TCDD (Fig. 5). Because this region also contains an AP-1 motif, a dyad symmetry element, and HRE-1, as mentioned above, the identified region is important in the transcription of the TH gene. Moreover, within this region, we narrowed the range over which TCDD activated AhR binding in an EMSA experiment. As shown in Fig. 6, sequence C (-238 bp to -213 bp) was shown to be an AhR-ARNT complex binding sequence. Although formation of the DNA-protein complex was inhibited by an XRE competitor, sequence C did not contain consensus XRE ([T/G]NGCGTG [A/C] [G/C]A; minimal consensus core sequence GCGTG) [17], contrary to the expectation.

Recently, several studies suggested that AhR binds regulatory sequences differently from the consensus XRE [42,43]. For example, the following sequences were identified: an AHRE-II sequence (CATG{N6}C [T/A]TG) in the promoter region of CYP1A2 and aldehyde dehydrogenase 3A2; an AnoC sequence ([C/T]GCG [C/T]GCGC [C/A]GC) in the promoter region of CYP1B1; and an XRE-like sequence ([G/T] [C/A]GGG) in the promoter region of PON-1 [42,44]. Because sequence C did not contain every sequence, we called sequence C, "AHRE-III".

TH gene expression induced by TCDD exposure would be regulated by AHRE-III mediated mechanism independent of the conventional AhR-mediated pathway.

Although XRE and other motifs are rarely found together in the promoter regions of the same gene, the mechanism of selection for AhR binding among multiple motifs was not investigated. According to the ontological characterization of AHRE-II-regulated genes, XRE-regulated, AnoC-regulated [43] and AHRE-II-regulated genes showed strong functional coherence with significant enrichment in ion channels and transporters, unlike XRE-regulated genes [43]. Selection of the binding sequence in promoter regions by AhR should be related to functional selection of AhR toxicity.

In order to investigate the relation of the AHRE-III-AhR-mediated pathway to cell function, we performed a genome-wide homology search with AHRE-III. One of the results of this search indicated that AHRE-III was found at -6769 bp of the 3'-flanking region of the Mash-2 gene (mammalian achaete-scute homologues). Mash-2 is a member of the bHLH transcription factor family, similar to AhR. Members of the bHLH family are related to neural development and differentiation and form a molecular signaling network. For example, Hes-1 (hairy and enhancer of split homolog 1) and Hes-5 downregulate Mash-1 and Math-1, respectively. Mash-1 and Math-1 subsequently downregulate NeuroD and Math-2, and finally regulate the expression of nerve-specific genes [45]. Hes-1, which is a key modulator in controlling neural proliferation and differentiation, was reported to be upregulated by AhR [46]. Mash-2 was also reported to be related to neurogenesis [47]. In the neural differentiation of P19 embryonal carcinoma cells, Mash-1 and Mash-2 were shown to regulate differentiation in a reciprocal manner. Mash-2 mRNA is specifically expressed in the early phase, whereas Mash-1mRNA is expressed in the late phase.

Therefore, we hypothesize that AhR could mediate a wide variety of genetic controls, including the detoxification process and neural development through transactivation, driven or not driven by XRE. The target pathway of AhR-mediated gene expression could be selected by cell types based on their functions and the molecular processes that characterize them.

Consequently, the AhR-AHRE-III-mediated pathway participates in neural development rather than xenobiotic metabolism or the detoxification process, and TCDD may induce neurodevelopmental disabilities through the AhR-AHRE-III-mediated pathway.

## Conclusion

The toxicity of TCDD in the nervous system has not been well studied. In this study, we suggest that TCDD directly regulates the dopamine system by TH gene transactivation via the AhR-AHRE-III-mediated pathway. The mechanism differs from the conventional AhR-XRE-mediated pathway and xenobiotic responses.

Concerning the neurotoxic phenotype of TCDD, this is the first evidence of a direct correlation between abnormal expression of the TH gene, which is an elementary process of dopaminergic dysfunction, and AhR-mediated genomic control transmitting TCDD action to the cells.

## Abbreviations

AhR: aryl hydrocarbon receptor; TH: tyrosine hydroxylase; ARNT: aryl hydrocarbon receptor nuclear translocator; TCDD: 2, 3, 7, 8-tetrachlorodibenzo-p-dioxin; XRE: xenobiotic response element; ADHD: attention deficit hyperactivity disorder; EMSA: electrophoretic mobility shift assay.

## Competing interests

The authors declare that they have no competing interests.

## Authors' contributions

EA designed the study and performed all experiments. SY was responsible for the HPLC experiment and EMSA. SU contributed to the EMSA experiment. MIS contributed to experimental design, participated in data analysis, and wrote the manuscript. All authors participated in the preparation of the final manuscript and approved the submission.
